# Role of procedure-specific consent forms in clinical practice: a systematic review

**DOI:** 10.1308/rcsann.2024.0079

**Published:** 2024-10-03

**Authors:** J Norvill, C Bent, JA Mawhinney, N Johnson

**Affiliations:** ^1^ Royal Chesterfield Hospital NHS Foundation Trust, UK; ^2^ East Sussex Healthcare NHS Trust, UK; ^3^ The Pulvertaft Hand Centre, University Hospitals of Derby and Burton NHS Foundation Trust, UK

**Keywords:** Procedure-specific consent forms, Consent form, Informed consent, Informed consent document

## Abstract

**Introduction:**

Consent forms play an active role in the consent process with generic, handwritten consent forms (GCF) often the standard across the National Health Service. Increasingly, procedure-specific consent forms (PSCF) are being used as an alternative. However, concerns remain about whether they meet the standard for consent. We therefore conducted a systematic review with the objectives of investigating evidence for PSCF, study methodology and medicolegal criteria.

**Methods:**

This systematic review was prospectively registered on PROSPERO (CRD42023392693) and conducted from 1 January 1990 to 17 March 2023 using the MEDLINE, Embase, CINAHL, CENTRAL and Emcare databases. A grey literature search was also performed. All studies evaluating PSCF in medical and surgical settings were included. Risk-of-bias analysis was performed using ‘RoB 2’ and ‘ROBINS-I’. Meta-analysis was not possible because of the results’ heterogeneity.

**Findings:**

We identified 21 studies investigating PSCF with no systematic reviews and meta-analyses reported. Most studies were quality improvement projects (*n* = 10) followed by randomised studies (*n* = 5). No definitive legal guidance for PSCFs and no studies assessing their role in litigation post-procedural complications were identified. PSCFs were associated with improved documentation (70%–100%; *n* = 11) and legibility (100%; *n* = 2) compared with GCF. Randomised studies (*n* = 4) investigating patient understanding and recall for PSCF were inconclusive compared with GCF.

**Conclusions:**

The heterogeneous evidence available merely demonstrates superior documentation of PSCF compared with GCF. Studies do not adequately investigate the impact on informed consent and fail to address the associated legal concerns. Further randomised studies with patient-centric outcomes and consideration for medicolegal criteria are needed.

## Introduction

Informed consent is an ethical and legal necessity for all procedures in healthcare.^1,2^ The Royal College of Surgeons of England (RCS England) states that for consent to be informed clinicians must be convinced that the patient has adequately obtained and understood information about their diagnosis, proposed treatment and its implications.^1^ Notably, the Montgomery ruling signifies a paradigm shift in best practice for informed consent. Clinicians must now take a patient-centric approach towards their decision making instead of the traditional paternalistic approach under the Bolam standard.^3^ The ruling’s importance is reinforced by the recent Independent Medicines and Medical Devices Safety review.^4^

Consent forms play an active role in the consent process by acting as a communication tool and documentation of patient–clinician discussions.^2^ Department of Health consent forms are used widely in the National Health Service (NHS). These forms are generic, non-standardised, handwritten documents.^5^ Unsurprisingly, the content of generic consent forms (GCF) is heterogeneous with strong variation in the procedure-specific complications and risks reported.^6^ They are also often inadequately completed and illegible.^7^ Importantly, however, such variation can be viewed positively because it allows for the consenting clinician to personalise their discussions with patients.

Increasingly, procedure-specific consent forms (PSCF) are being used as an alternative to GCF. PSCF may help standardise the consent process and improve communication with patients through the use of accurate, legible, preprinted information about a proposed intervention.^6^ They may also help protect clinicians against rising NHS litigation cases through robust consent documentation.^8,9^ Significantly, 4% of all general/vascular surgical negligence cases settled by the Medical Defence Union from 1990 to 2000 involved consent issues. Poor documentation was cited as one of several recurring themes in litigation cases.^10^

PSCF are not widely used in the UK and are not endorsed by Department of Health.^5^ A survey of surgical consultants in the UK found that only a minority to use PSCF. In such instances, most PSCF were produced locally rather than nationally.^11^ Concern remains that their widespread use will lead to clinicians not fully explaining procedures and their associated risks to patients.^7^ If true, the use of PSCF would harm the consent process and likely increase the number of litigation cases.

In light of the Montgomery ruling, questions remain as to whether PSCF are a viable alternative to GCF and in keeping with our modern understanding of informed consent. On this basis, we performed the first systematic review evaluating the role of PSCF in clinical practice.

The objectives of this systematic review were to:

• report the methodology used in studies evaluating the use of PSCF in clinical practice;
• assess the evidence for the use of PSCF in clinical practice;
• consider the need for further studies attesting to their use including appropriate methodology;
• consider legal criteria for the use of PSCF in clinical practice.

## Methods

Our systematic review adhered to the Preferred Reporting Items for Systematic Reviews and Meta-Analyses (PRISMA) guidelines.^12^ The study protocol was prospectively published on PROSPERO (CRD42023392693).

### Search strategy

The search strategy was devised, peer-reviewed and performed by an experienced clinical librarian. The following databases were searched: MEDLINE, CINAHL, HMIC, CENTRAL, Embase and Emcare to identify studies reporting outcomes for PSCF. Searches were from 1 January 1990 to 17 March 2023. Languages were unrestricted. General search terms included:

• (Pre-printed consent form* OR procedure-specific consent form*)
• (Pre-printed consent form* OR procedure-specific consent form* OR procedure-specific complication*)
• (Pre-printed consent form* OR procedure-specific consent form* OR procedure-specific risk*)
• (Pre-printed consent form* OR procedure-specific consent form* OR procedure-specific document*).

A manual grey literature search of NHS trust policies, dissertations, theses and charity publications was performed using the same criteria.

### Inclusion criteria

All studies whose consent process for a procedure used a PSCF across both medical and surgical disciplines were included. Studies were conducted after 1 January 1990 and languages were unrestricted. Every effort was made to obtain English language translations for foreign language papers. Only full-text manuscripts and risk-of-bias assessments were included in the review. When conference publications were obtained, all efforts were made to obtain a full-text manuscript. All prospective and retrospective studies including randomised and non-randomised interventional trials, observational studies, cohort studies, quality improvement projects and audits of adult and paediatric populations were included.

### Exclusion criteria

All studies investigating only generic and/or handwritten consent forms were excluded. Their references were, however, evaluated for further relevant publications. Editorials, case reports, book chapters and literature not presenting primary research on PSCF were also excluded.

### Risk-of-bias (quality) assessment

The ‘RoB 2 Cochrane risk-of-bias tool’ for randomised trials was used to assess bias of randomised trials and their respective methodology. Studies were graded high risk, some concerns or low risk.^13^ The ‘ROBINS-I: Cochrane risk-of-bias tool’ for non-randomised studies of interventions was used to assess bias of non-randomised studies and their respective methodology. Studies were evaluated at preintervention, intervention and postintervention.^14^

### Data extraction

**Figure 1:**
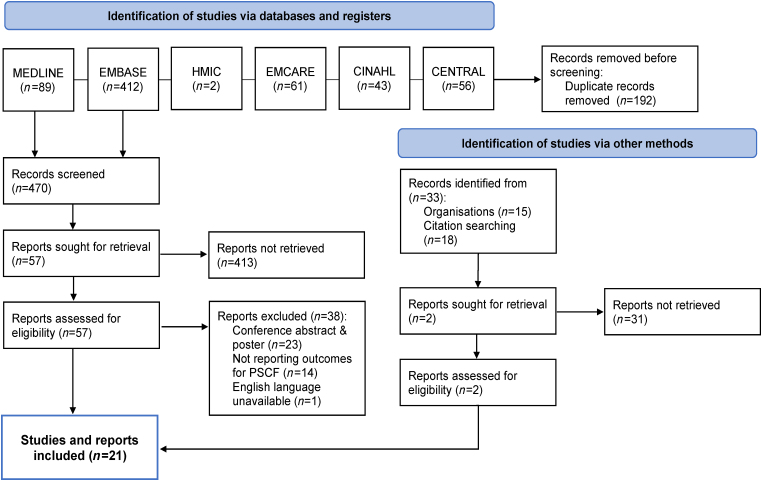
PRISMA flowchart of studies assessed for inclusion. PSCF = procedure-specific consent form. Adapted from Page *et al*^12^

Studies were collated and ordered in Rayyan^©^. Three reviewers (JN, CB and JM) each independently screened studies. Figure 1 shows the PRISMA inclusion flow chart.

Study titles and abstracts were initially reviewed by each of the three authors followed by assessment of the full text. Subsequently, JN and CB independently extracted studies’ data and assessed for risk of bias. Throughout the process, any eligibility disputes were resolved by achieving group consensus at review meetings.

The following data were extracted: country, medical or surgical speciality, procedure, grade of consenting clinician, study design, number of patients, patient demographics, number of centres, data collection method and validation, length of study, intervention, comparator, primary and secondary outcomes, study results, type of PSCF, procedure risks and complications from relevant surgical body and legal opinion.

### Data synthesis

Studies were summarised and key characteristics extracted. Data were synthesised qualitatively and quantitatively where appropriate. Substantial heterogeneity in the studies was expected, therefore, a meta-analysis was not performed. However, where possible, risk or odds ratios with 95% confidence intervals for comparative outcomes were extracted.

## Findings

### Study selection

A total of 695 studies were generated from the search of databases and grey literature (Figure 1). After removal of duplicates, 503 studies were eligible for screening. Twenty-one studies met the criteria for inclusion in the systematic review and risk-of-bias assessment (Table 1).

**Table 1:** Summary of studies reporting outcomes for procedure-specific consent forms

Study	Speciality	Procedure	Study design	Patient demographics	Intervention and comparator	Primary outcomes	Key results^+^
Study outcomes: Documentation and legibility							
Tan *et al* (2017)^15^ UK	Surgical*:* Ophthalmology, urology, obstetrics and gynaecology	Circumcision and laparoscopic tubal occlusion, cataract surgery, laparoscopic nephrectomy	Retrospective case notes review: Review of patient notes; *n* = 60; 1 NHS trust	N/A	Cataract surgery and nephrectomy PSCFs vs circumcision and tubal occlusion GCFs	Legibility and documentation of procedure risks	PSCFs 100% adherence GCFs ‘less favourable’ with variable documentation
Issa *et al* (2006)^16^ USA	Surgical: Urology	Standard and laser TURP; retropubic and perineal RP	QIP^++^ Review of consent forms and patient survey; *n* = 204; 1 centre	N/A	iMedConsent™ for TURP and RP vs written urological consent forms (GCF)	Documentation, content and quality of GCF	Suboptimal documentation with GCF: procedure benefit missing (4.4%) and deficient (22.6%); inconsistent risk documentation (0%–96%) Patient (96.1%) preference for iMedConsent™ vs GCF
Broussard *et al* (2009)^17^ USA	Medical: Paediatric anaesthetics	Paediatric procedural sedation	QIP Review of anaesthesia charts; *n* = 84; 1 centre	Mean age, 43–71 months	Preprinted packet including order set, consent form and sedation monitor form vs standard practice	Documentation of consent forms, procedure notes, sedation monitor form pre- and postintervention Order form compliance pre- and postintervention	Increased documentation of consent form post- vs preintervention (*n* = 30, 71% vs *n* = 13, 31%; *p* < 0.001)
Isherwood *et al* (2013)^18^ UK	Surgical: Orthopaedics	THR	QIP Standardised data collection sheet; *n* = 100; 1 centre	N/A	Procedure-specific complication sticker for THR vs GCF	Documentation of procedure risks	PSCF associated with improved documentation of procedure-specific risks vs GCF (86%–100% vs variable; *p* < 0.0001)
Crozier *et al* (2018)^19^ Australia	Surgical: General surgery	Laparoscopic cholecystectomy	Two-arm study phase two: prospective RCT Review of consent forms; *n* = 62, 1 centre	N/A	Phase two: PSCF for laparoscopic cholecystectomy vs GCF	Phase two: Documentation of procedure complications	PSCF associated with improved documentation of complications (93%–97% vs 49%–91%; *p* < 0.05) except for bleeding and infection vs GCF
Bajada *et al* (2017)^20^ UK (Wales)	Surgical: Orthopaedics	Elective and acute orthopaedic procedures	QIP Review of consent forms; *n* = 140; 2 centres	N/A	Focused surgeon in training teaching and procedure-specific complications and risks stickers vs all Wales GCF	Documentation and legibility of procedure complications and risks	Intervention group associated with increased legibility (100% vs 25%), complications listed (100% vs 62%) and consent form offered to patients (38% vs 0%) vs GCF Surgeons preferred PSCF to GCF
St John *et al* (2017)^7^ UK	Surgical: Breast surgery (phase two)	Mastectomy, breast-wide local incision (phase two)	Two phase QIP Review of consent forms; *n* = 189; 2 NHS trusts	N/A	Phase two: OpInform.com breast surgery consent forms (PSCF) vs handwritten consent form (GCF)	Legibility, accuracy and documentation	PSCF associated with increased documentation of complications (100% vs 44%) and no domain failures (0% vs 10–30%) vs GCF
Khan *et al* (2018)^21^ UK	Surgical: General surgery	Groin hernia repair	Retrospective audit with patient questionnaire Review of patient records and patient postal questionnaire; *n* = 115; 1 centre	Median age, 60 years; Sex*, 1:19	Preprinted procedure-specific consent stickers vs handwritten GCF	Documentation of procedure benefits, choice, LA, procedure complications and discharge summary	Stickers associated with increased documentation of procedure benefits (100% vs 47%; *p* = 0.0001), complications (100% vs 37%; *p* = 0.0001), discharge summary (100% vs 37%; *p* = 0.0001) and reduced LA choice (4% vs 18%; *p* = 0.045), vs GCF
Owen *et al* (2018)^22^ UK	Surgical: Orthopaedics	THR and TKR	QIP Review of consent forms; *n* = 112; 1 centre	N/A	PSCF for TKR and THR vs handwritten GCF	Legibility, documentation and consistency	PSCF associated with increased legibility (100% vs 78%) and risk documentation (100% vs variable) vs GCF
Prado and Waring (2019)^23^ UK	Surgical: Orthodontics	All orthodontic procedures	QIP Review of consent forms; *n *≥ 100; 2 centres	N/A	PSCF for orthodontic treatment vs GCF ± photography consent form	Number 5 of consent forms used Documentation of appliance type, patient and clinician information, treatment duration and risks	PSCF associated with increased consent form use (100% vs 82%) vs GCF PSCF associated with increased documentation of appliance types (100% vs 95%), duration (88% vs 34%) and risks (100% vs variable) vs GCF
Singh *et al* (2020)^24^ UK (Wales)	Surgical: Orthopaedics	Surgical repair of traumatic hip fracture (emergency cases)	QIP Retrospective review of patient records; *n* = 74; 1 centre	N/A	Targeted surgeon in training teaching ± procedure risks stickers for hip fracture surgery vs GCF	Impact of teaching ± stickers on abbreviations, offering consent form copy and documentation	Cycle 1 (*n* = 26) targeted teaching: reduced use of abbreviations (38% to 20%), increased documentation of procedure risks (31% to 72%) and offering of consent form copy (12% to 48%) Cycle 2 (*n* = 24) teaching + stickers*:* reduced use of abbreviations (7%), static documentation of procedure risks (67%), fewer consent forms offered to patients (8%)
Study outcomes: Patient understanding							
Finch *et al* (2009)^25^ UK	Surgical: Urology	TURP	Randomised trial Patient questionnaire; *n* = 100; 1 centre	Mean age, 72–75 years	BAUS PSCF for TURP vs conventional Department of Health consent form (GCF)	Patient understanding of procedure risks and benefits after consent	No difference in patient understanding of likelihood of improved symptoms (median, 80%; *p *> 0.05) and patients’ median estimation of risk of complications PSCF patients more accurately predicted the risk of reoperation vs GCF (median, 30% vs 10%; *p* = 0.007)
Barritt *et al* (2010)^26^ UK	Surgical: Orthopaedics	KA and TKR	QIP Patient questionnaire; *n* = 160; 1 centre	N/A	OrthoConsent^®^ PSCF for KA and THR + standard yellow form vs standard yellow consent form (GCF)	Patient understanding of procedure	OrthoConsent^®^ + GCF associated with improved patient understanding of KA and THR vs GCF (KA, 81% vs 57%, *p* < 0.01; TKR, 82% vs 58%, *p* < 0.01)
Hall *et al* (2012)^27^ USA	Surgical: General surgery	Cholecystectomy and inguinal herniorrhaphy	Cohort study Patient questionnaire (23-item consent comprehension questionnaire; trust in physician scale); *n* = 75; 1 centre	Mean age, 56–61 years; Sex, 8:15	iMedConsent™ for cholecystectomy and inguinal herniorrhaphy vs preintervention standard practice	Patient understanding of procedure risks, benefits and alternatives	iMedConsent™ associated with improved patient understanding of risks, benefits and alternatives vs preintervention (60% vs 50%; *p* < 0.001) No difference in ambivalence, trust or anxiety pre- vs postintervention Patient preference for SDM (98% vs 71%, *p* = 008)
Arnander *et al* (2013)^28^ UK	Surgical: Hand surgery	CTD	Cohort study Patient interview post-consent, before surgery; *n* = 80; 1 centre	Mean age, 55–60 years; Sex, 1:2	OrthoConsent^®^ PSCF for CTD 2 weeks before surgery + on-the-day GCF vs on-the-day GCF	Patient understanding of complications, alternatives and satisfaction with consent	OrthoConsent^®^ associated with increased awareness of alternatives (93% vs 43%; *p* < 0.001), risk recall (mean, 7 [range 5–8] vs 4 [range 0–8]; *p* < 0.001) and consent satisfaction (mean, 9.4 [range 8–10] vs 5.3 [range, 4–9]; *p* < 0001) vs control group
Krishnamoorthy *et al* (2016)^29^ UK	Surgical: Cardiothoracic surgery	Elective or urgent CABG	Randomised study using a pre-/post-test design Patient questionnaire before consent or surgery and post-surgery; *n* = 100; 1 centre	Mean, 66 years; Sex, 1:5	PSCF for CABG vs Department of Health consent form (GCF)	Patient understanding of procedure, risks and benefits Satisfaction with consent	PSCF associated with increased patient identification (62% vs 30%, *p* = 0.011) and understanding of procedure (66% vs 20%, *p* = 0.001) vs GCF PSCF associated with improved patient understanding of benefits (72% vs 8%, *p* < 0.001) and risks (82% vs 10%, *p* < 0.001) vs GCF PSCF associated with improved consent satisfaction vs GCF (94% vs 85%, *p* < 0.001)
Baker *et al* (2021)^30^ UK	Surgical: Ophthalmology	Retinal detachment repair surgery	QIP Patient questionnaire; *n* = 50; 1 centre	N/A	PSCF for retinal detachment repair surgery vs GCF	Patient understanding of consent form and procedure risks	PSCF associated with increased consent form knowledge (76% vs 56%; *p* = 0.0357), risk explanation (49% vs 23%; *p* = 0.001), and patient wishes documentation vs GCF (33% vs 9%; *p* < 0.00001)
Study outcomes: Patient recall							
Clark *et al* (1991)^31^ USA	Medical: Anaesthetics	GA for elective procedure	Cohort study Patient interview 4–6 weeks after surgery; *n* = 233; 1 centre	Not reported	Anaesthesia consent form (PSCF) vs GCF	Patient recall of procedure risks	GCF recalled more anaesthetic risk information vs PSCF (33% vs 19%; *p* < 0.01)
Pomeroy *et al* (2021)^32^ Ireland	Surgical: Orthopaedics	THR	Prospective study using a post-test-only control group (random allocation) Patient phone interview 4 weeks after surgery; *n* = 60; 1 centre	Not reported	PSCF for THR vs GCF	Patient recall of risk of surgical complications 4 weeks after surgery	PSCF associated with increased number of risks recalled vs GCF (mean, 1.43 vs 0.67; *p* = 0.0131) No significant difference between groups for consent satisfaction (mean, 9.5 vs 8.8; *p* = 0.3063)
Power *et al* (2022) ^33^ Ireland	Surgical: Orthopaedics	THR (unilateral)	RCT Patient phone interview 4 weeks after surgery; *n* = 70; 1 centre	Not reported	Preadmission consent document including PSCF vs GCF	Patient recall of risk of surgical complications 4 weeks after surgery	Poor recall in both groups (16% vs 13%; *p* = 0.49) 30% of patients did not recall a single risk. Subgroup analysis excluding ‘non-responders’ reported increased recall rate in both groups (24.5% vs 18.25%, *p* = 0.02)
Studies outcomes: Other							
Hall *et al* (2015)^34^ USA	Surgical: General surgery	Cholecystectomy and inguinal herniorrhaphy	Cohort study Database of iMedConsent™ written information and clinic patient–provider audio-recordings; *n* = 37; 1 centre	Mean age, 56 years; Sex, 1:20	iMedConsent™ for cholecystectomy or inguinal herniorrhaphy vs audio-recordings	iMedConsent™ information vs patient–provider discussions	Meetings discussed 37% (95% CI, 0.07–0.67) and 33% (95% CI, 0.21–0.43) of information found on iMedConsent™ cholecystectomy and herniorrhaphy forms Information not stated in iMedConsent™ was identified (herniorrhaphy, 20 items and cholecystectomy, median 27.5 items)

BAUS, British Association of Urological Surgeons; CABG, coronary artery bypass graft; CTD, carpal tunnel decompression; DoH, Department of Health; GA, general anaesthesia; GCF, generic consent form; KA, knee arthroscopy; LA, local anaesthetic; NHS, National Health Service; PA, physician’s assistant; PSCF, procedure-specific consent form; QIP, quality improvement project; RP, radical proctectomy; SDM, shared decision making; TURP, trans-urethral resection of the prostate; THR, total hip replacement; TKR, total knee replacement; +Key results, includes results of primary outcomes and where possible results of secondary outcomes. Only results relevant to PSCF and GCF are reported; ++QIP, included quality improvement projects, single/multiple audit cycles and closed-loop audits. *Female to male ratio, approximations were used when appropriate

### Study characteristics

#### Demographics

Most studies investigating PSCF were conducted in the UK (*n* = 13) and from surgical specialities (*n* = 19) (Table 1 and Appendix 1 – available online). Orthopaedics (*n* = 7) was the most frequently cited specialty. Total hip replacement (*n* = 6) was the most frequently investigated PSCF. Nine studies referenced a surgical body for their PSCF risks and complications. A copy of the PSCF was not always available to view. Most studies (*n* = 14) used a paper-based PSCF. Other PSCF included preprinted stickers (*n* = 4) and digital media (*n* = 3). When reported (*n* = 14), doctor in training was the most common grade of consenting clinician. These studies lacked specific details about the consenting clinician and often cited various grades. Only one study reported using data validation.

#### Design

Most studies identified (*n* = 10) were quality improvement projects (QIP). Five randomised studies were identified.^19,25,29,32,33^ All randomised studies compared paper-based PSCF vs GCF. GCF included handwritten, Department of Health consent forms and a generic, unspecified consent form.^19,25,29,32,33^ All patients were randomised into groups in a non-blinded manner by computer-based programs and/or sealed envelopes containing a consent form.^19,25,29,32,33^ All studies except Pomeroy *et al* were clearly defined as randomised. In this study, patients were randomised as part of a post-test-only control group design.^32^

### Evidence assessing PSCF in clinical practice

PCSF were generally assessed in terms of documentation, legibility, patient understanding and patient recall of procedure-specific risks and complications.

#### Documentation and legibility

Thirteen studies assessed documentation and/or legibility by reviewing patients’ medical records and the content of their consent forms (Table 1).^7,15,16,18–24^ Most studies identified were QIP (*n* = 8). One randomised study was identified.^19^ All 13 studies associated PSCF with improved documentation (70%–100%; *n* = 11) and legibility (100%; *n* = 2) vs GCF.^7,15,16,18–24^

#### Patient understanding

Six studies assessed patient understanding by patient questionnaires and/or interviews using standardised question proformas (Table 1).^25–30^ Five studies associated PSCF with improved patient understanding of the procedure, its benefits and risks/complications.^26–30^ The randomised trial by Finch *et al* reported inconclusive results with no significant difference in patients’ understanding of likelihood of improved symptoms (*p* > 0.05) and estimation of risk of complications (*p* > 0.05).^25^ However, PSCF patients more accurately predicted the risk of reoperation at 10 years vs GCF (*p* = 0.007).^25^

#### Patient recall

Three studies assessed patient recall by patient interviews using standardised question proformas (Table 1). Studies showed inconclusive results with Clark *et al* reporting significantly lower patient recall of anaesthetic risks for PSCF vs GCF (*p* < 0.01). Power *et al* reported poor recall of risks in both intervention and GCF groups (*p* = 0.49). Conversely, Pomeroy *et al* reported a significantly higher number of risks recalled with PSCF vs GCF (*p* = 0.0131).

#### Legal criteria

Three studies obtained legal advice for their consent form (Table 1).^19,20,26^ Of these, no study provided details of the advice obtained. No studies assessed the role of PSCF in litigation proceedings. No definitive legal guidance for their use in clinical practice was identified.

### Risk of bias and quality of evidence

#### Randomised studies

Five studies were included in the RoB 2 assessment. Most randomised studies (*n* = 3) had ‘some concerns’ (Appendix 2 - available online and Appendix 3 - available online). Pomeroy *et al* was the only low-risk randomised study identified. All studies were low risk in the domains: selection of the reported result and missing outcome data. The domains ‘measurement of the outcome’ and ‘randomisation process’ were least likely to be identified as low risk.

#### Non-randomised studies

A total of 16 studies were included in the ROBINS-I assessment. Most studies (*n* = 9) were identified as ‘moderate risk’ (Appendix 4 - available online and Appendix 5 - available online). Singh *et al* and Isherwood *et al* were the only low-risk studies identified. All studies were low risk in the domain: deviation from intended interventions. No studies, including individual domains, were identified as ‘critical risk’ of bias. The domain, confounding variables, was least likely to be identified as low risk.

### Conference abstracts

A total of 23 conference abstracts reporting outcomes for PSCF were identified. Results are comparable to studies included in this systematic review and are summarised in Appendix 6 - available online.

## Discussion

Informed consent is an ethical and legal necessity for both patients and clinicians.^1,2^ The standards for consent have shifted substantially since the Montgomery ruling.^3,4^ There is now increased scrutiny on consent practice in both medical and surgical settings.^1,2,4^ It is therefore essential that the use of PSCF is scrutinised and satisfies our modern standard of consent. We present findings of the first systematic review evaluating the role of PSCF in clinical practice.

### Evidence assessing PSCF in clinical practice

#### Documentation and legibility

Twenty-one studies were included in this review. In most instances, doctors in training were responsible for obtaining consent with documentation and legibility (*n* = 13) the most frequently reported study outcomes.^7,15,16,18–24^ Positively, the improved documentation associated with PSCF is likely to support less-experienced doctors in training through functioning as a memory aid.^18^ Significantly, however, improved documentation does not necessarily translate into an improved consent process for patients. Regardless of grade, clinicians still need to engage in meaningful dialogue and fully explain procedures to patients. PSCF should never serve as a superficial ‘tick-box’ exercise for clinicians.

Relevantly, Hall *et al* investigated whether consent discussions corresponded with the documentation on PSCF. Their study revealed only 37% and 33% of information present on iMedConsent^™^ cholecystectomy and herniorrhaphy consent forms was discussed with patients.^31^ In addition, relevant points neglected in PSCF were identified from discussions.^31^ These data indicate that consent forms may be less important to the consent process than we previously thought.

In short, data suggest that improved documentation alone is unlikely to benefit informed consent. More appropriate outcomes such as patient understanding and recall may be more meaningful.

#### Patient understanding

Five studies associated PSCF with improvements in patient understanding of a procedure, its benefits and complications/risks compared with GCF.^26–30^ Unfortunately, the clinical applicability of these studies is limited because no studies were identified as low risk of bias. In these studies, patients’ level of education and prior knowledge of a procedure was often not measured. Notably, patients who have previously undergone a procedure or attained higher education may be quicker to understand and retain the procedure complications/risks explained during consent.

Arnander *et al* was the only study identified that excluded patients who had previously received a procedure for which they were being consented. OrthoConsent^®^ was associated with improved knowledge of treatment alternatives (*p* < 0.001), risk recall (*p* < 0.001) and consent satisfaction (*p* < 0001) compared with GCF. However, these findings are interpreted with caution because this study was identified as having ‘serious concerns’ in our risk-of-bias assessment. Concerningly, all patients were consented on the day of surgery using a GCF. Patients in the intervention group received a postal PSCF two weeks before surgery.^28^

In short, further studies which truly investigate patient understanding while adequately controlling for confounders are needed to confirm superiority of PSCF vs GCF.

#### Patient recall

Two randomised controlled trials (RCTs) and one cohort study assessed patient recall. Their findings are inconclusive.^31–33^ Pomeroy *et al* was the only RCT identified as low risk of bias. Patients consented with a PSCF reported a significantly increased number of risks recalled compared with GCF (1.43 vs 0.67; *p* = 0.0131).^32^ However, overall retention of risks recalled was generally low in both groups.^32^ Other studies reported no difference in risk recall between groups, and greater recall of risks with GCF vs PSCF.^31,33^

Notably, these studies only investigated patient recall with PSCF in the initial weeks post-surgery. To adequately understand their impact on patient recall, data collected up to one to two years post-surgery is likely needed. Our findings may also reflect broader issues associated with consent forms including their complexity, use of jargon and patient readability issues.^35^ To mitigate these issues, consent forms should be used with accompanying consent tools.

In short, data investigating patient recall for PSCF are inconclusive. Clinicians should use various accompanying consent tools such as videos, diagrams and patient leaflets when using PSCF.^4,35^

## Legal and regulatory aspects

### Legal evidence

Currently, there is no definitive legal guidance attesting to PSCF in the consent process. No studies investigating PSCF in medicolegal proceedings are available. Few studies (*n* = 3) briefly cited legal input for their consent form.

Relevantly, we identified an editorial by Cooper *et al* (2017) that did not meet our inclusion criteria. However, it was particularly relevant because the authors developed eight PSCF for which they received medicolegal input. They concluded that consent always needed to be personalised to the patient and use of PSCF should never replace patient–provider discussions.^8^ As per RCS England guidance, personalisation of PSCF is readily achieved through the use of free-text boxes.^1^

In short, the medicolegal benefits of PSCF are not proven and sufficient supporting evidence is not available.

### National guidance

PSCF are not recommended by the Department of Health and RCS England.^1,5^ Nevertheless, several NHS trusts continue to recommend and actively use them.^36–40^ Notably, University Hospitals Plymouth NHS Trust recommends PSCF combined with patient information leaflets as the ‘gold standard’ for informed consent.^38^ Again, trusts' highlight the need for free-text space to allow for personalisation of ‘material’ risks to the patient.^38,40^

### Digital consent

Digital-based consent forms are increasingly being used worldwide. In the USA, iMedConsent™ application was developed for members of the American College of Surgeons and received positively.^16,27,34^ In the UK, OpInform.com and OrthoConsent^®^ allow clinicians to print-off computer-generated PSCF.^7,26,28^ Innovatively, Concentric Health’s e-platform allows for paperless consent that can be personalised to the patient.^41–43^ Clinicians in the NHS and worldwide need to be ready for digital consent and supported by high-quality evidence attesting to its use in clinical practice.

### Limitations

Limitations of this study focus on the heterogeneity of the studies reported. The substantial variation in consent forms identified and outcomes reported makes direct comparisons challenging. Regardless, our robust review adheres to PRISMA guidelines and provides valuable insight into the quality of evidence available for PSCF.^12^

### Future studies

Additional randomised studies that investigate PSCF using patient-centric outcomes are warranted. Given the nature of consent forms, we recognise that blinded randomised trials are difficult to perform. However, confounders such as previous surgery, prior knowledge, education and number of clinic visits should always be controlled for in studies. Pertinently, patient-centric outcomes including patient understanding, recall and satisfaction should be prioritised. Tools measuring shared decision making should also be considered (e.g. SDM-9 and CollaboRATE).^44,45^

## Conclusions

Most studies investigating PSCF merely highlight their improved documentation compared with GCF. Those that investigated patient understanding and recall are inconclusive and affected by bias. Concerningly, no studies assessing their perceived medicolegal benefits were identified.

In conclusion, if we are to truly implement the Montgomery ruling in clinical practice, studies investigating consent should prioritise patient-centric outcomes. The perceived notion that improved documentation equates to an improved consent process for patients is inaccurate. To further optimise consent, studies should also consider the medicolegal evidence for PSCF and aim to understand their role alongside accompanying consent tools.
